# Examining the variation in consent in general surgery

**DOI:** 10.1308/rcsann.2023.0020

**Published:** 2023-05-23

**Authors:** A Sebastian, L Wyld, JL Morgan

**Affiliations:** University of Sheffield Medical School, UK

**Keywords:** Consent, Surgery, Litigation

## Abstract

**Introduction:**

Consent is a fundamental aspect of surgery and expectations around the consent process have changed following the Montgomery vs Lanarkshire Health Board (2015) court ruling. This study aimed to identify trends in litigation pertaining to consent, explore variation in how consent is practised among general surgeons and identify potential causes of this variation.

**Methods:**

This mixed-methods study examined temporal variation in litigation rates relating to consent (between 2011 and 2020), using data obtained from National Health Service (NHS) Resolutions. Semi-structured clinician interviews were then conducted to gain qualitative data regarding how general surgeons take consent, their ideologies and their outlook on the recent legal changes. The quantitative component included a questionnaire survey aiming to explore these issues with a larger population to improve the generalisability of the findings.

**Results:**

NHS Resolutions litigation data showed a significant increase in litigation pertaining to consent following the 2015 health board ruling. The interviews demonstrated considerable variation in how surgeons approach consent. This was corroborated by the survey, which illustrated considerable variation in how consent is documented when different surgeons are presented with the same case vignette.

**Conclusion:**

A clear increase in litigation relating to consent was seen in the post-Montgomery era, which may be due to legal precedent being established and increased awareness of these issues. Findings from this study demonstrate variability in the information patients receive. In some cases, consent practices did not adequately meet current regulations and therefore are susceptible to potential litigation. This study identifies areas for improvement in the practice of consent.

## Introduction

Consent is a core component of good surgical practice, defined as the act of gaining permission from an individual prior to receiving any form of treatment, examination or investigation.^1^ According to the General Medical Council (GMC), there are seven principles of consent^1^ (Table 1) and the Royal College of Surgeons of England guidelines describe ten steps in the process of consent^2^ (Table 2). The RCS England states that when consenting patients prior to elective procedures, the following topics should be discussed: the diagnosis and prognosis, what the procedure entails, who will be involved in the procedure, why the intervention is necessary, the chance of success, associated risks and complications, and the alternative options that are available.^3^ Consent is also a process often involving several conversations and the majority of these issues are covered well before obtaining the consent form signature.

**Table 1:** General Medical Council’s seven principles of consent

Principle no.	
1	All patients have the right to be involved in decisions about their treatment and care and be supported to make informed decisions if they are able
2	Decision making is an ongoing process focused on meaningful dialogue: the exchange of relevant information specific to the individual patient
3	All patients have the right to be listened to, and to be given the information they need to make a decision and the time and support they need to understand it
4	Doctors must try to find out what matters to patients so they can share relevant information about the benefits and harms of proposed options and reasonable alternatives, including the option to take no action
5	Doctors must start from the presumption that all adult patients have capacity to make decisions about their treatment and care. A patient can only be judged to lack capacity to make a specific decision at a specific time, and only after assessment in line with legal requirements
6	The choice of treatment or care for patients who lack capacity must be of overall benefit to them, and decisions should be made in consultation with those who are close to them or advocating for them
7	Patients whose right to consent is affected by law should be supported to be involved in the decision-making process, and to exercise choice if possible

**Table 2:** The Royal College of Surgeons of England’s ten steps of consent

**Step**	**Task**
1	Explain the diagnosis to the patient. Ensure that the information is given in a format the patient can understand. Explain the prognosis if untreated
2	Explain the options for treatment. Explain the risks and benefits of various treatment options side by side and ensure that not having any treatment is included among the options. Describe the likelihood of success of the various options and the impact that treatments will have on the patient’s life
3	Explain the consent and decision-making process. Ensure the patient understands that they are expected to make a supported decision and their rights within this process. Do not assume that the patient will be familiar with the concept of supported decision making and check whether they have a supporter
4	Time for deliberation. Surgeons should allow sufficient time for patients to deliberate on available options and to consider their goals and wishes in terms of their treatment. This may include reading further information or accessing online resources to provide them with more information
5	Discuss the patient’s wishes, needs, views and expectations. It is important not to make assumptions regarding what a ‘good’ outcome from treatment would look like for the patient. Different patients will have different life priorities and different views regarding what the best available outcome might be or what risks are acceptable to them
6	Discuss trade-offs with the patient. Explain how different options will or will not achieve their goals and any potential impact that the options will have
7	Provide any relevant information not already covered. If there is further information that would have a bearing on the decision that the patient is being asked to make that has not already been discussed and/or understood by the patient, then these factors should be explained. This is of particular importance in cases in which the process has spanned a period during which changes may have occurred in the patient’s condition or around the risks and benefits of available treatment options
8	Has the patient understood? It is imperative that the person seeking consent is satisfied that the patient has understood the information that they have been given and that any decision they make will be made independently and from an informed position
9	Respect the patient’s decision
10	Signing of the form and maintaining a decision-making record. The consent form should be signed at the end of the discussion, provided the patient has made a decision. The patient should be given a copy of the form to review and retain. Details of the discussion and copies of any information given to the patient should be included in the patient’s notes

The Montgomery vs Lanarkshire Health Board case (2015)^4^ is a landmark supreme court ruling that has significantly changed the model of consent from the traditional paternalistic approach to a more shared decision-making process. Multiple studies demonstrate that not all surgeons are aware of the Montgomery ruling and recent changes have met with mixed attitudes.^5–7^ Although most clinicians agree it is a positive change, some have felt it has added to the responsibilities of doctors in an already time-pressured environment. Doctors are now required to identify and discuss risks that are materially significant to the patient regardless of how unlikely they may be; this can often prove challenging in practice owing to the limited time spent with patients and the subjective nature of material risks.^8,9^ The literature shows that the consent process is not always as comprehensive as recommended by current regulations, with key information frequently omitted from the discussion and the materiality of risks not always taken into consideration.^5,10^ Issues with consent are a common source of litigation in surgery. The impact that the Montgomery ruling may have on rates of litigation relating to consent is discussed widely in the literature, but a detailed review of trends within general surgery has not been performed previously.^11,12^

This study aimed to explore temporal changes in consent-related litigation before and after the Montgomery ruling, study variation in how consent is practised among general surgeons and identify the potential sources of this variation.

## Methods

Research ethics approval was obtained on 20 August 2022 from the University of Sheffield Research Ethics Committee (reference 043108). A mixed-methods study design was used to explore the issue of consent in general surgery, including three components:

1.  Analysis of changes in the rate of consent-related litigation in the UK before and after the Montgomery ruling in 2015.
2.  Qualitative semi-structured interviews with health professionals regarding the consent process.
3.  A questionnaire survey of a larger group of health professionals about the consent process.

A mixed-methods synthesis was then performed to interpret the results and draw conclusions.

### Litigation rate impacts of the Montgomery ruling

Litigation data were obtained from National Health Service (NHS) Resolutions, the department that manages and keeps a record of medicolegal claims faced by the NHS, via a Freedom of Information request (2000) on 30 July 2021.^13^ Data were reported as the number of claims received by NHS Resolutions per year and the number and cost of claims closed per year. The percentage of litigation cases related to consent, and the total costs of closed cases incurred by the NHS, were calculated for each financial year for 5 years before and 5 years after the Montgomery ruling in 2015 (from 2011 to 2020). Chi-squared analysis was used to identify any statistically significant differences in the absolute number and proportion of cases related to consent, before and after 2015. The temporal trend was also derived.

### Qualitative interviews with healthcare professionals

#### Population

Semi-structured qualitative interviews were conducted with general surgeons purposively selected for varying levels of experience (from Foundation Year 1 to experienced consultants) and across a range of different subspecialties (e.g., colorectal, breast, upper gastrointestinal and vascular surgery) within a single UK health region.

#### Recruitment

Recruitment took place between January and June 2022. Participants were approached by personal contact by the study lead or by direct email. No reminders were sent if emails were not answered.

#### Interviews

Written informed consent was obtained. Individuals were interviewed in-person, by video or by phone, and were given the interview schedule prior to the interview date.

The interview schedule was developed by the study team based on the published literature and expert opinion of the topic from within the study team. Iterative modification took place and new issues of relevance were raised as interviews progressed.

Topics of interest included: understanding of informed consent, risk management, consent process, training on consent and ways to improve current practice.

Interviews ceased once saturation of the themes occurred. Interviews were recorded, transcribed verbatim and the Framework approach was used for data analysis.^14^

### Questionnaire survey

#### Questionnaire design and validation

A survey questionnaire was subsequently developed, based on the above interview findings (to give content validity), to explore how participants would document consent when presented with the following case vignette.

A 38-year-old female presents with a 2-day history of right iliac fossa pain and raised inflammatory markers. She has had an ultrasounds scan which revealed a small amount of free intraperitoneal fluid but was non-diagnostic. A clinical diagnosis of appendicitis has been made and you have been asked to consent the patient for a laparoscopic appendicectomy.

Participants were asked to document the name of the proposed treatment, the intended benefits, risks and complications, any additional/alternative interventions that may become necessary during the course of the treatment and any additional information they would like to document.

The survey instrument was not formally piloted before use within the study because it followed the format of the standardised NHS consent form used to document surgical consent in standard clinical practice and therefore could be assumed to have face validity, acceptability, useability and comprehension.

#### Population

Potential participants included anyone who had previously consented patients for laparoscopic appendicectomy or worked in a position in which they were required to perform this task. The survey was developed using the Google Forms electronic survey platform and was sent to consultants, core surgical trainees and higher surgical trainees (in general surgery) currently working in South Yorkshire hospitals. Access to the survey was also advertised nationally via social media platforms (Facebook and Twitter) and the White Rose, iBRA-NET (implant Breast Reconstruction evaluation-NETwork) and East Midlands North surgical rotation collaborative groups. The questionnaire recorded the subspecialty interests of each participant and their current training grade.

#### Sample size

The survey was exploratory and descriptive, and therefore no specific sample size was calculated, but the size of the survey was set to allow the results to be generalisable to the wider UK population of consultants and surgical trainees (multicentre, different trust sizes and designations, a range of training grades, specialities). The platform used to create and distribute the survey could not record the number of people that accessed the link, therefore the response rate could not be calculated and therefore how representative the sample size was of the general population cannot be calculated.

### Statistical analysis

Descriptive statistics are presented as means and standard deviations for normally distributed data and median and range for non-normally distributed data. Comparative analyses were performed using chi-squared test. Statistical analyses were performed in IBM SPSS statistics version 25. A *p*-value < 0.05 was considered significant.

## Results

### Litigation rate impacts of the Montgomery ruling

#### General claims environment

**Figure 1:**
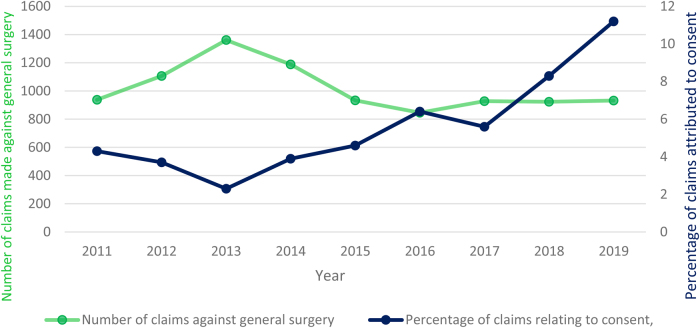
Total volume of claims made against general surgery and the proportion of cases attributed to issues with consent

The litigation data from NHS Resolutions includes all cases of litigation related to general surgery registered between 2011 and 2020, during which time the total number of claims made against general surgery has been fairly stable. Figure 1 demonstrates this trend and shows a slight decrease in the total number of settled claims after 2015 (Figure 1).

**Figure 2:**
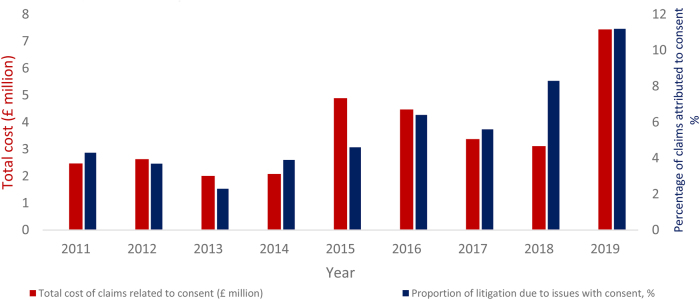
Proportion of litigation due to issues with consent (%) and the cost incurred to the NHS (£ million)

#### Claim relating to consent issues

Analysis demonstrated that the percentage of claims related to informed consent more than doubled over the period 2011 to 2020 (Figure 1). There was also a disproportionate rise in medicolegal cases relating to consent after 2015, with the proportion of claims owing to consent consistently higher in every year following 2015 compared with any point in the preceding half decade. The average percentage of cases related to consent during 2011–2015 was 3.61% (201/5,562) but during 2016–2020 it had risen to 7.91% (287/3,629), amounting to a 118% increase (*p* < 0.001). The total amount paid by the NHS for claims related to consent has also seen a dramatic rise since 2015 (Figure 2); from £14.07 million (2011–2015) to £25.18 million (2016–2020). 

### Interviews

Thirteen surgeons were invited to participate in the qualitative interviews and 12 agreed to be interviewed (response rate 92.3%). Interviews ceased after 12 participants due to saturation of themes being reached. Participant characteristics are shown in Table 3. Surgeons from across England were recruited, with participants included from the following regions: Yorkshire and Humber Deanery, Northern, Kent, Surrey and Sussex, and Wessex. The average duration of the interviews was 59min. Five primary themes were identified and these are shown in Table 4 with exemplar quotes.

**Table 3:** Training grade, subspeciality and gender of participants

**Interview participants**				**Survey respondents**		
**Participant**	**Training grade**	**Subspeciality**	**Gender**	**Training grade**	***N* = 86**	**%**
1	Consultant	Upper gastrointestinal	Male	Consultant	24	27.9
2	Registrar	Colorectal	Male	Middle grade	46	53.5
3	Consultant	Colorectal	Male	Foundation Year 2	16	18.6
				**Subspeciality**	***N* = 86**	**%**
4	Post-certificate of completion of training registrar	Colorectal	Female	Colorectal	41	47.7
5	Post-certificate of completion of training registrar	Hepatobiliary	Female	Breast	9	10.5
6	Registrar	Breast	Female	Upper gastrointestinal	10	11.6
7	Consultant	Breast	Male	Hepatobiliary	5	5.8
8	Consultant	Colorectal	Male	Emergency general surgery/trauma	6	7.0
9	Registrar	General	Male	Transplant	1	1.2
10	Foundation Year 2	General	Male	Endocrine	1	1.2
11	Foundation Year 2	General	Male	General surgery/core training	12	14.0
12	Consultant	Trauma	Male	Did not answer	1	1.2

**Table 4:** Representative quotes from each interview theme

Subtheme	Representative quote
Theme 1: Understanding of informed consent	
Definition	Those details should include the benefits of those treatments, potential risk of those treatments and any potential alternatives to those treatments, including doing nothing. [Participant 6; female; post-certificate of completion of training registrar]
Montgomery	To tell the patient the risks and benefits and potential alternatives that the patient thinks are important. [Participant 2; female; registrar]
Views on current guidance	What a reasonable patient should know, that’s very hard to define, so it really does open up the scope that you should really discuss an infinity amount of risk … that doesn’t necessarily tally with how much time is allocated. [Participant 7; male; consultant]
Theme 2: Risk	
Risk selection	My approach is to basically talk about anything and everything. I go [through] everything that’s common and everything, everything through to worst case scenarios pretty much. [Participant 11; male; Foundation Year 2] [I]f there’s a 0.05%, I won’t mention that because that by the law of probabilities, if you operate it will not occur in the lifetime of you operating. [Participant 12; male; consultant]
Explanation of risk	[R]isk of this 2% that means if 100 people had the operation the odds are 2 of those people would likely get that complication. [Participant 11; male; Foundation Year 2]
Common bile duct injury	[T]hey [patients who suffer a common bile duct injury] die earlier; we know that. Do we tell them? No, we say bile duct injury, I think it’s the confidence that I’m happy. I cut the bile duct once. [Participant 1; male; consultant]
Stoma risk (uncomplicated appendicitis)	I think [if the presentation is] more sinister and CT [computed tomography] findings suggest there might be more to it, then I would consent for a stoma. [Participant 3; male; consultant] [E]very single appendix for every patient, so that’s part of the process, so I consent for bowel resection, I consent for stoma formation. [Participant 2; male; registrar]
Checking understanding	This isn’t an exam for the patient … as long as you’ve explained to them in a methodical manner, and they’ve asked questions going along, well that’s it. They’ve understood. [Participant 12; male; consultant]
Risk of death for low-risk cases	I put risk to life, or death, on every consent because the majority of operations we do are under GA [general anaesthesia], which is probably like 1 in 10,000 risk. [Participant 9; male; registrar] I think anybody who consents an appendix or a perianal abscess in a young person for death maybe shouldn’t be doing surgery. [Participant 12; male; consultant]
Risk perception	[W]hat’s happened to you in your life will make you more or less risk averse. Sometimes I think people are born more or less risk averse. [Participant 6; female; post-certificate of completion of training registrar]
Theme 3: Consent process	
Consent form	They act as a bit of an aid memoire to the doctor of what risks they put down, but they’re certainly not very educational to the patient. [Participant 7; male; consultant]
Preprinted labels/stickers for consent forms	[W]e have stickers for all of our colorectal sections … think they are really useful. It means, it should mean that everybody is consenting them in a standardised way. [Participant 4; female; post-certificate of completion of training registrar]
Timing of consent	I hate consenting people on the day …. they aren’t sure they want to go ahead and they don’t feel they can say no, because they’re there and everything is set up ready to go. [Participant 4; female; post-certificate of completion of training registrar]
Theme 4: Training	
Personal experience	[S]urgery is an apprenticeship. You get taught by the good trainers. Some trainers are good, some trainers aren’t. [Participant 12; male; consultant]
Delegation	If they [trainees] are knowledgeable enough about procedure, that they can reasonably anticipate answering any questions. [Participant 8; male; consultant]
Inappropriate	[I remember my] first house job where I would go and consent people for an Ivor Lewis oesophagectomy. [Participant 8; male; consultant]
Theme 5: How to improve current practice	
Training	[Y]ou clearly can’t teach people to consent for every single individualised procedure, but the basic principles, basic tenants of consent we’ve done, and there are courses out there of course, but pretty easy to do. [Participant 3; male; consultant] If you do prescriptive training, where you become a chimpanzee, ok, you get taught how to do it. You’re no longer a clinician. You’re a person who follows flow charts. Anybody can follow a flow chart, but the art lies in making it bespoke for everybody. [Participant 12; male; consultant]
Additional resources	Preprinted, pre-electronically done, consent form, it can save us [time] and help the patient … do we have to remember all the percentages … It is a lot of numbers. [Participant 1; male; consultant]
E-consent	[e-consent] massively enhances that process because you’ve got all the potential risks and benefits that you probably should be discussing and then you can sit down and easily tweak it for the patient. [Participant 12; male; consultant]

#### Theme 1: Understanding informed consent

The interviews demonstrated variations in opinion on what is required for informed consent and how this should be practised. Not all participants were fully aware of the Montgomery ruling or the guidance from the RCS England on informed consent. Many felt that the current guidance and expectations were difficult to achieve in clinical practice, mainly owing to time constraints.

#### Theme 2: Risk

There was variation in how participants handled the concept of risk in their consent consultations. Some felt that all risks were potentially “material”, some felt that low-risk events were such low probabilities that they were not worth disclosing. Participant varied in how they explained risks to patients, although many used the analogy of a number out of 100 individuals, rather than percentages. There was a general feeling that the concept of risk is not conveyed to patients in a standard way during the consent process.

#### Theme 3: Consent process

There was variation in how the consent process was performed, with some surgeons utilising preoperative consent clinics and others consenting on the morning of elective surgery. Some surgeons used preprinted stickers or forms and a minority used electronic consent systems.

#### Theme 4: Training

There was a general feeling that the training participants had received in the process of informed consent was highly variable and not always adequate, especially for high-risk major procedures. There was variation in opinion on who should lead the process of informed consent and when it should be delegated.

#### Theme 5: How to improve current practise

Several ideas to improve the current system were mentioned, including formal taught courses as part of the training programme for junior surgeons, and the use of electronic consent packages.

### Survey

In total, 86 participants completed the questionnaire (Table 3 shows participant demographics). The survey did not record the number of people who accessed the link; therefore, the response rate could not be calculated. The mean number of potential risks mentioned was 11.3 ± 3.5 (range 4–23). The most commonly documented risks were bleeding (85/86; 98.8% of participants), infection (83/86; 96.5%), risk of damage to surrounding organs/structures (83/86; 96.5%) and risk of venous thromboembolism (70/86; 81.4%). Table 5 shows the risks and complications that were documented and how frequently they were mentioned.

**Table 5:** Risks and complications explained/mentioned during consent

Risk/complication	Number (%) of participants that documented complication on consent form (*N* = 86)
Bleeding	85 (98.8)
Infection	83 (96.5)
Pain	55 (64.0)
Damage to surrounding organs/structures	83 (96.5)
Scar/wound problems	40 (46.5)
Hernia	55 (64.0)
Collection/abscess	30 (34.9)
Seroma	2 (2.3)
Anaesthetic risk	40 (46.5)
VTE/blood clots	70 (81.4)
Death	20 (23.3)
MI/heart attack	17 (19.8)
CVA/stroke	6 (7.0)
Adverse drug reactions	4 (4.7)
COVID risk	30 (34.9)
Respiratory problems (including LRTI)	25 (29.1)
Adhesions	13 (15.1)
Ileus	8 (9.3)
Stumpitis	6 (7.0)
Stump leak	9 (10.5)
Conversion to open	56 (65.1)
Bowel resection	56 (65.1)
Stoma	45 (52.3)
Gynaecological intervention	55 (64.0)
Salpingo-oophorectomy	23 (26.7)
Ovarian cystectomy	7 (8.1)
Insertion of drains/catheters	31 (36.0)
Blood transfusion	20 (23.3)

CVA = cerebrovascular accident; LRTI = lower respiratory tract infection; MI = myocardial infarction; VTE= venous thromboembolism

The specificity of risks also varied between participants. For example, 46.5% of participants (40/86) consented for “anaesthetic risks” but some participants specified what these risks were: 29.1% (25/86) recorded respiratory problems and 4.7% (4/86) of participants discussed adverse drug reactions. Similarly, 64.0% (55/86) of participants stated there was a generic risk of “gynaecological complications” (or need for gynaecological referral), whereas some went on to record specifically what this might involve: 26.7% (23/86) included a chance of salpingo-oophorectomy and 8.1% (7/86) recorded the possibility of ovarian cystectomy, with only one (1.2%) participant documenting the risk of potential impact of future fertility.

On average, consultants recorded significantly fewer risks and complications compared with trainees; with trainees documenting 3.6 times more risks than consultants (95% confidence interval 2.0–5.2; *p* < 0.05). The risk of “stoma formation” in particular was reported more frequently among core trainees (CTs) and middle grades than consultants; with 86.7% (13/16) of CTs and 61.4% (28/46) of middle grades documenting the risk compared with 13.6% (3/24) of consultants (*p* < 0.001). “Pain” was also disclosed more often by junior trainees; being referenced by 100% (16/16) of CTs compared with 71.7% (33/46) of middle grade participants and 25.0% (6/24) of consultants (*p* < 0.001).

Most of the risks were mentioned fairly similarly across different subspecialties. The possibility of stoma formation was mentioned by 52.3% (45/86) but was more commonly documented by hepatobiliary (HPB) surgeons (5/5; 100%). The risk of bowel resection followed a similar trend, being mentioned by 100% (5/5) of HPB surgeons, which was considerably higher than the group average of 65.1% (56/86), although overall this variation was less than with stoma formation. Risk of death was recorded by 24.7% (20/81) of all respondents and when reviewed according to specialty, HPB surgeons again recorded the possibility of death most frequently at 80% (4/5), followed by colorectal surgeons (10/41; 24.4%) and general trainees (3/12; 25.0%). However, none of these differences reached statistical significance.

In addition to risks and complications, participants were asked what additional information they would discuss when consenting this patient, and the following topics were identified: the possibility of negative appendicectomy/incorrect diagnosis (mentioned by 41/86; 47.7%), option of antibiotic therapy or conservative management (mentioned by 12/86; 14.0%), preoperative computed tomography scans (mentioned by 8/86; 9.3%) and the potential need for further surgery (discussed by 20/86; 23.3%). No differences were identified between different training grades or subspecialties in regard to what additional information was discussed.

## Discussion

The data illustrate a significant increase in litigation related to consent in the post-Montgomery era, suggesting varying levels of compliance to the new guidelines among general surgeons. Although the Montgomery ruling did not introduce any new legislation, it has led to a change in GMC guidelines,^1^ which govern how consent is practised in the NHS, and clinicians who are not up to date with this legal precedent may be more vulnerable to litigation.

The key change introduced by the Montgomery ruling is the recommendation of discussion around material risks. Material risks include any risk that a reasonable individual would consider important or is significant to that particular individual, regardless of how unlikely that risk may be. Clinicians have the responsibility to identify such risks and it can be difficult to judge what risks are important to an individual, particularly for very uncommon risks. This makes the adequacy of consent much more subjective, and the outcomes of medicolegal cases related to consent less predictable, attributed to the variability in how courts assess material risk.^12,15^ During the interview phase, many participants pointed out that the guidelines offer very little practical advice on how to efficiently identify material risks, and given that various subjective factors (such as profession, lifestyle, hobbies) can influence the materiality of a risk, it can be difficult to identify, particularly when clinicians have limited time with patients.

The survey data show the practice of consent is highly variable and, in some circumstances, does not meet what is required by current guidelines. Only 14.0% (12/86) of survey respondents stated that they would discuss other treatment options (such as antibiotic therapy) despite current guidelines emphasising that reasonable alternate treatment choices must be discussed with all patients.^1–3^ During the interview phase, some participants felt that risks below a certain level of incidence do not need to be disclosed, on the basis they will not perform enough surgeries in their career to encounter such rare events and disclosing such risks will only cause unnecessary anxiety for the patient. This is in contrast to the new guidelines stating that there are no arbitrary cut-off points and any risk the patient is likely to attach significance to must be discussed. In the case of Spencer vs Hillingdon Hospitals NHS Trusts (2015) the risk of pulmonary embolism (as stated by the defendant) was 1/50,000 and the court concluded it should have been discussed with patients, showing that probability alone should not determine the significance of a risk.^10^

Previous experiences with complications were shown to have a considerable influence on whether it was included in the consent process and the manner in which it was discussed. For example, common bile duct injury is a rare (around 1/300) but significant complication associated with laparoscopic cholecystectomy. Interviewed individuals who had experience of working with patients who have sustained such complications and seen the life-changing impacts it can have (such as HPB surgeons) were more likely to be open with patients about the full implications and severity of this complication. An individual’s past experiences seemed to impact their perception of certain complications, which may ultimately affect how patients perceive these risks and the choices they make. Similarly, consultants documented fewer complications than trainees. In the interviews, consultants were more likely to omit certain complications from the conversation on the basis they had never experienced them before, and they had greater confidence that they are unlikely to happen. Junior trainees may, therefore, be more risk averse than their more senior colleagues, or there may be an underlying difference in the way junior trainees are being trained as a result of ongoing changes in the climate of consent.

The materiality of stoma risk could not be assessed from the case vignette but, regardless, 52.3% (45/86) still documented this risk in the survey. The risk of stoma formation was reported more frequently by junior training grades than consultants, corroborating the findings from the interviews which showed that junior trainees were more likely to routinely discuss the possibility of stoma formation, whereas consultants and senior trainees preferred to discuss it for select cases where they felt there was evidence of increased risk. Senior participants were reluctant to discuss the risk of stoma formation for uncomplicated appendicitis because it can be a source of anxiety and patients may refuse surgery based on a low-probability event. However, if a patient would rather opt for less-effective treatments because they cannot contemplate the possibility of certain risks, then that choice should be discussed, because those risks are clearly material to the patient. There were no significant differences in the documentation of bowel resection (or colectomy) between training grades, suggesting that surgeons may be more reserved in reporting the possibility of stoma formation owing to how emotive it can be, and the impact on patients’ risk perception.

For the majority of the participants their training in consent was mostly experiential and self-directed, with very little assessment or feedback from senior colleagues, resulting in highly varied learning experiences among trainees. Given the increased complexity of consent law after the Montgomery ruling, and the associated increases in litigation, improving the quality of training in this area may be imperative in supporting trainees to practise consent in accordance with current regulations and ultimately protect them from litigation. Informed consent training programmes are effective in improving knowledge of the fundamental principles of consent, documentation of consent and confidence in carrying out this task.^16,17^ Some participants debated that consent should continue to be learnt by observing others, like other aspects of surgery. However, this method of learning is heavily dependent on supervisors, and many participants stated they did not receive the supervision or feedback necessary to have adequate training.

E-consent may be another valuable resource to reduce variation in consent because documents available via the digital consent platform usually automatically include a baseline number of risks associated with a procedure but allow the opportunity for clinicians to modify and highlight risks, to tailor the process to each individual patient. This improves the efficiency of the process and means surgeons are not required to memorise all the risks and associated incidence rates. The form can be accessed by patients at a later date and the programme also contains information regarding the procedure (e.g., leaflets, visual aids) to facilitate patient understanding and allow them to be more informed. Digital forms have been shown to improve consistency of the information provided to patients and are associated with significantly lower omission of core risks, fewer errors, as well as enhanced legibility of the information.^18^ The limitations of implementing e-consent on a large scale are yet to be identified, but are likely to include increased costs. However, e-consent has the potential to be a valuable adjunct and may help develop the practice of consent.

### Study limitations

The NHS Resolution data are limited by the dates the claims were filed, which do not reflect when they occurred or when they were settled. In addition, the cost of claims relate only to those claims that are closed, and not to those ongoing. This affects the conclusions that may be drawn from these data because some claims may not be filed or closed for several years after the incident occurred.

Purposive sampling used in the interview phase of the study is prone to researcher bias compared with probability-based sampling and therefore vulnerable to errors in judgement by the researcher,^19^ although the larger sample recruited by the survey study helps to compensate for this. There is also potential volunteer bias because those who expressed an interest in participating may have different characteristics from the general surgical population, and this therefore reduces the external validity of the data set findings.

The response rate of the survey could not be calculated, and how representative the sample size is of the wider population is therefore unknown. The representation of different subspecialties varied considerably, and although this represents the distribution of subspecialties within general surgery, some subspecialty groups were significantly smaller than others. As a result, formal comparisons could not be made between certain groups owing to inadequate power. Power calculations were not performed because the study was exploratory and there were no assumptions or expectations regarding variation between subspecialties.

## Conclusion

In summary, this study demonstrated that there is considerable variation in the practice of consent, the ideologies regarding how it should be practised and surgeons’ attitudes towards current guidelines surrounding consent. The findings show the quality of information provided by surgeons is inconsistent and, in some instances, does not adequately satisfy what is required by current regulations in a time where litigation for problems with consent are on the increase in general surgery. This study has helped to identify areas for development that could improve quality of consent and benefit both clinicians and patients.
